# Improving breast and colorectal cancer screening information through co-design with underserved groups in an urban area

**DOI:** 10.1186/s12875-026-03522-z

**Published:** 2026-08-19

**Authors:** Olga C. Damman, Loebna Sabbah, Danielle R.M. Timmermans, Abanoub Iskander, Ingeborg S. van Strien-Knippenberg

**Affiliations:** 1 Department of Public & Occupational Health, Amsterdam UMC location VUmc, Amsterdam, the Netherlands; 2 Quality of Care program, Amsterdam Public Health research institute, Amsterdam, Netherlands

**Keywords:** Cancer screening, Disparities, Health literacy, Underserved populations, Public involvement, Co-design, Information provision, Informed decision-making, Health inequality

## Abstract

**Background:**

Certain groups participate less in Dutch population-based breast cancer (BC) and colorectal cancer (CRC)-screening, such as people with lower socio-economic status or ethnic minorities in deprived urban areas. We aimed to investigate the perspective and underlying needs of underserved groups regarding information provision about BC- and CRC- screening, and to design novel information strategies accordingly. The ultimate aim was to reach them more effectively and support informed decision-making.

**Methods:**

In total, 28 co-design sessions were conducted in deprived neighborhoods of Amsterdam, the Netherlands, for four backgrounds separately, i.e., Moroccan, Turkish, Surinamese, and no migrant background. We performed 16 sessions with women (*N* = 44; 4 sessions per group; 40–78 yrs) about BC- and CRC-screening, and 12 with men (*N* = 28; 3 sessions per group; 54–79 yrs) about CRC-screening. Creative assignments, summaries, and field notes were analyzed using thematic analysis.

**Results:**

Three overall themes were identified. First, there was very limited knowledge about BC and CRC and the idea of screening for these cancers, which made it difficult for people to relate cancer screening information in general to existing knowledge structures. Second, people were in an early stage of the decision-making process, with positive but largely uninformed attitudes towards BC- and CRC-screening. Third, although participation in cancer screening was generally considered desirable, it was not a priority in daily life. Especially people with a migrant background emphasized that invitations via post were easily missed/forgotten. Co-designed novel information strategies included group information sessions in local communities, short awareness/information flyers, and audiovisual information in multiple languages.

**Conclusions:**

People from underserved populations recognized the importance of BC- and CRC-screening in taking care of their health. However, findings suggested that some key information did not come across, due to a mismatch between information content typically provided -focusing on cancer screening- and basic knowledge and information needs of the participants. Additionally, there was a mismatch between typical information delivery (i.e. post) and how participants said to handle information in their daily lives. Co-designed strategies can be utilized by screening organizations.

**Supplementary Information:**

The online version contains supplementary material available at https://doi.org/10.1186/s12875-026-03522-z.

## Background

Cancer screening rates in the Netherlands are declining, with participation rates of 68% for Colorectal Cancer (CRC)-screening and 71% for Breast Cancer (BC)-screening in 2022 [1]. These rates are falling close to or just below the European targets strived for, i.e. 65% for CRC-screening and 75% for BC-screening. Just like in other countries [2–4], certain subgroups do not participate equally, mostly people from ‘underserved groups’, i.e., those with lower Socio Economic Status (SES) and from racial/ethnic minorities in urban areas [5–7]. People from underserved groups are generally at increased risk of cancer and cancer-related mortality, due to several risk factors (e.g., unhealthy lifestyle, worse self-management skills) [8, 9], or, in the case of BC specifically, at increased risk of diagnosis at younger age and with unfavorable tumor characteristics [10]. Many people with lower SES have lower Dutch proficiency and health literacy (HL) [11], which can hinder their understanding of cancer screening information. Reducing existing disparities in access to cancer screening and in understanding information is therefore of great importance.

One key aspect of many cancer screening programs, for example in Western and Northern Europe, is to enable informed decision-making (IDM) about participation [12, 13]. This generally means understanding the purpose of the program and its benefits and harms, weighing benefits and harms, and taking one’s values into account in decision-making [12]. IDM is considered key because not all people who participate also individually benefit from screening. Harms such as false positive results, unnecessary worry, and exposure to unnecessary tests and treatments, are intrinsic to cancer screening and should ideally be considered against expected benefits (i.e., reduced cancer mortality) [12, 14]. Benefit/harm information is experienced as complex by the lay public and at times as inconsistent with previous beliefs [15–19]. This problem is likely exacerbated for people from underserved populations [20, 21]. The benefit/harm ratio of cancer screening may in some cases also differ for people from underserved populations, depending on risk factors [8, 9].

The literature describes barriers to IDM among people from underserved groups at different interactive levels [20, 21], i.e. lacking knowledge [22–24], certain beliefs about cancer and cancer screening [7, 24], information miscomprehension [19, 25–28], defensive information processing [16, 24], difficulties with interacting with the healthcare system [26, 28], and financial/logistical barriers (e.g., competing demands, scheduling difficulties) [22, 26]. HL, ‘cancer screening literacy’ or ‘cancer prevention literacy’ are concepts suggested to play a key mediating role in this interplay [28–30], referring to problems in information access and processing, decision-making, and implementing decisions. However, what exactly the more underlying, latent needs are among people from underserved groups in making more informed decisions, and how we can support them in making such decisions, remains unknown.

To actually improve strategies to reach and inform people from underserved groups, it is important to collaborate more extensively with the target groups themselves. This is particularly important for understanding latent needs that are likely more difficult to identify using interviews or surveys. Stokholm et al. [31] noted that few studies investigated cancer screening invitees’ own views on what is relevant knowledge about cancer screening. A suitable approach to investigate invitees’ own perspectives is co-design. This approach focuses on research-to-action, emphasizes direct engagement of local priorities and perspectives, and yields findings that directly reflect a community’s needs and concerns [32, 33]. This approach places emphasis on collaborative creativity and design of concepts, products, or services. Co-design is thus often defined as any act of collaborative creativity, shared by two or more people [34].

In recent years, several studies used participatory methods in the context of cancer screening disparities, but these studies have focused on increasing participation and not specifically on information provision and IDM. For example, educational programs, specific videos emphasizing the importance of cancer screening, and reminder interventions were co-designed [35–37]. Although some of these interventions were effective in terms of greater uptake, they are mostly not applicable in the Dutch population-based BC- and CRC-screening context (see Table 1). This is because interventions such as reminder invitations are seen as too persuasive and/or not serving the aim of IDM. In addition, counseling by health professionals is not possible in the Netherlands, because they do not have a role in BC- and CRC-screening. Dutch citizens are invited per post and participate at local screening centers of the Dutch center for population screening -BC-screening-, or via a self-test -CRC-screening- (Bevolkingsonderzoek Nederland). The first time that invitees thus see a healthcare professional in the screening context is after the decision has been made. For women participating in BC-screening this is a mammographer, in CRC-screening a healthcare professional is only seen in case of a referral to the hospital (see Table 1).

**Table 1:** Short description of the Dutch BC-and CRC-screening programs

Breast cancer screening: The current Dutch population-based BC-screening program is designed for women between 50 and 75 years of age. Once every 2 to 3 years, women in this age group are invited for a mammography. The aim is to detect breast cancer at an early stage, when the prospects for successful treatment are greatest. Invitees for BC-screening receive an invitation and information leaflet (in Dutch, with links to information in other languages) through written post. Women have to make an appointment themselves, at a screening center in their neighborhood. The screening (i.e. mammography) takes place in that screening center by trained mammographers. The X-rays of the two breasts are sent to two radiologists for assessment. Women receive a letter with the result within 10 working days. If an abnormality is found, women are referred to secondary hospital care for further examination, i.e. an additional mammography or ultrasound scan. In many cases, a small bit of tissue breast is then also taken, i.e., biopsy.	
Colorectal cancer screening: The current Dutch population-based screening program for CRC-screening is designed for everyone between 55 and 75 years of age. Once every 2 years, people in this age group are invited. The aim is to detect colorectal cancer at an early stage – before the first symptoms appear, when the prospects for successful treatment are greatest. Invitees for CRC-screening receive an invitation and information leaflet (in Dutch, with links to information in other languages) through written post. A FIT (Fecal Immunochemical Test) is included as a self-test. The first step is to perform the self-test at home. People have to send the stool test in the enclosed envelope via the letterbox. Next, the stool is examined in a laboratory to assess if there is blood in the stool. People receive a letter with the result within 2 weeks. If blood is found in the stool, people are referred to secondary hospital care for further examination. This usually includes the follow-up test of a colonoscopy.	

One previous study did focus on information provision, rather than persuasion towards participation, in CRC-screening, in co-creation with people from underserved populations [38, 39]. Findings indicated, however, that information-oriented interventions (e.g., easy-to-read leaflets providing the essential information about screening or narrative information) had no effects on the socio-economic gradient of uptake or overall uptake, whereas an enhanced reminder approach did [39]. Although highlighting key information is arguably important for IDM, we need additional approaches that help people from underserved populations navigate the complex information landscape they are exposed to. Therefore, this study aimed to investigate the perspective and underlying needs of people from underserved populations in the Netherlands regarding information provision, and to design novel information strategies accordingly.

## Methods

### Design

This study used a co-design approach, guided by the principles outlined by Sanders & Stappers [34], and took place in local community centers in deprived neighborhoods in the Amsterdam region, the Netherlands (i.e. neighborhoods with the largest portions of low-income households). Overall, the approach was to first get to know the target group and understand their needs and barriers, and then work towards iterative design of novel strategies. The study was also guided by a citizen science board, consisting of intermediaries between the researchers and people from underserved groups. Members were, for example, a community worker, a welfare worker and a plain language specialist working for a magazine designed for people with a low income/living on a tight budget. The citizen science board was created by the research team specifically for our research project and did not exist before the project. The board first worked with us on the study reported in the current paper, and continued working with us on another study on cervical cancer screening. After that study, the board was disbanded. All members were reimbursed for their time through a volunteer allowance. The citizen science board helped our team to set priorities, design recruitment strategies and assignments for the co-design sessions, and to interpret results. Some members were also present during co-design sessions as translators and intermediaries to build trust. If citizen science board members were not available to translate during the sessions, we collaborated with other translators. Sessions with women concerned both BC- and CRC-screening and consisted of four sessions per group, conducted between November 2023 and February 2024. Sessions with men concerned CRC-screening only and consisted of three sessions per group, conducted between May 2024 and July 2024.

### Participants

Target participants were those between 50 and 75 years of age (women) and between 55 and 75 years of age (men), representing the age groups invited for BC-screening (50–75 yrs) and CRC-screening (55–75 yrs), who were from ‘underserved populations’ in deprived neighborhoods in Amsterdam, the Netherlands. We used the following working definition of ‘underserved groups’: people *without a migrant background and a very low educational level*, and people *with a non-Western migrant background* (Moroccan, Turkish, Surinamese) *and a low or medium educational level*. The three migrant populations were selected because these are the largest migrant groups from outside Europe in the Netherlands [40]. We did not set strict inclusion criteria, given the nature of community groups and participatory methods.

Participants were recruited through a mixture of approaches set out with the citizen science board. First, we contacted community workers and volunteers in local community care in targeted neighborhoods and asked if we could organize sessions together in local community centers. Second, we disseminated flyers in Dutch, Moroccan-Arabic, and Turkish in local community centers. Third, for the female groups, we disseminated a video in which two female citizen science board members explained the study and invited women to participate. This video and digital versions of the flyers were also disseminated through social media by the citizen science board and in neighborhood app groups.

**Fig. 1:**
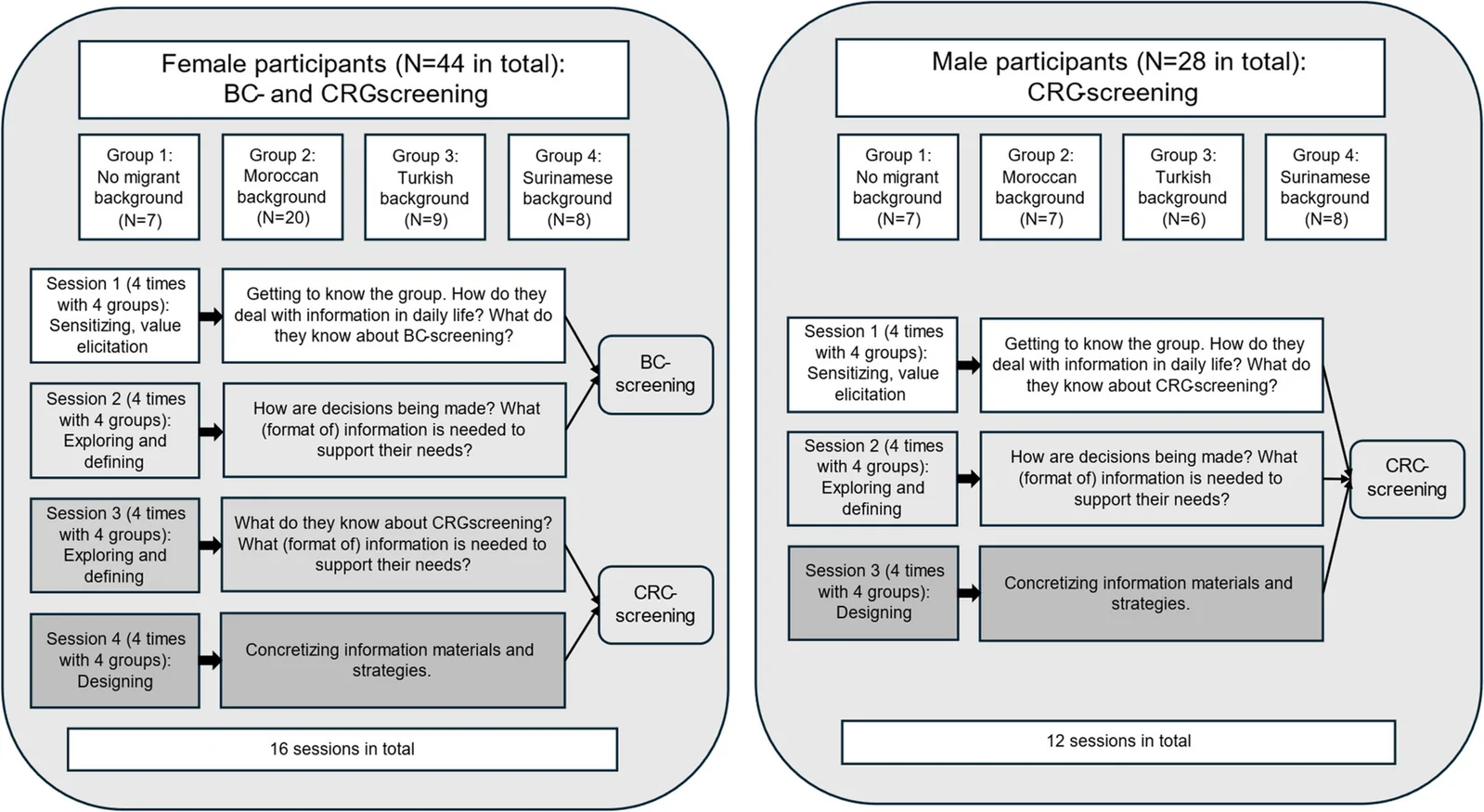
Overview of all sessions with all participants

All sessions were conducted separately for men and women due to the sensitivity of the topics and intimate parts of the body, and with groups of different backgrounds separately: Moroccan, Turkish, Surinamese, and no migration background. Figure 1 presents an overview of all sessions with all participants.

There were four sessions with each female group (the same women participated in the four sessions), resulting in 16 sessions in total, and three sessions with each male group (the same men participated in the three sessions), resulting in 12 sessions in total.

### Procedure and materials

If people showed interest, we sent them information about the study purpose in the way they preferred: written materials via post or e-mail, but in most cases audio messages through WhatsApp, first via intermediaries (e.g., community workers/volunteers at community centers) and in a next step via the researchers. Co-design sessions were held in local community centers in the following Amsterdam neighborhoods: Nieuw West, Zuid-Oost, and Noord. In some community centers, sessions were held prior or after existing group meetings, for example, an existing group of women with a Turkish background who met each Wednesday for breakfast. Other interested women with a Turkish background, who were not part of the existing group, could also participate. In other community centers, we rented a room specifically for a ‘new’ group of participants who did not know each other beforehand. All sessions were organized together with citizen science board members, local community workers/volunteers, and we provided food and drinks. Participants filled out informed consent before starting the first session.

A protocol was developed for each session, with PowerPoint presentations for general explanations. For women and men of Turkish background, these were translated into Turkish, because initial sessions with them indicated that they had much difficulty with reading Dutch. During the sessions, we also used written materials from the Dutch national BC- and CRC-screening as examples, and we designed creative assignments and templates. Supplementary file 1 provides the key assignments and materials used in the different sessions. All sessions lasted 2 h.

Session 1 in both female and male groups was focused on getting to know the target group and identifying key values in daily life as well as related to health. We used draft personas (Supplementary file 1, assignment 1) and asked participants to create those personas with us, focusing on priorities and frustrations in daily life, communication preferences and key information sources used. In addition, we prompted participants with key individual values (Supplementary file 1, assignment 2) and asked them to prioritize. We also had a group discussion about how participants dealt with information from various sources, and prompted them with an envelope with an invitation for BC-screening (female groups). Finally, we discussed what participants already knew about BC-screening (female groups) and CRC-screening (male groups), to capture first overall associative knowledge.

Session 2 in both female and male groups explored and defined how health-related decisions are usually made by participants, what the existing knowledge was about BC-screening (female groups) and CRC-screening (male groups) and what information would be needed to support their needs. Assignments (Supplementary file 1, assignment 3) included templates to be completed in pairs about the decision-making process and which thoughts, feelings, information, and opinions from others might influence the decision. We also encouraged discussions about potential benefits and harms of participating in the specific screening programs. Finally, we discussed different existing and newly created visuals for the BC-and CRC-screening programs in the Netherlands (Supplementary file 1, assignment 4, only in female groups) to stimulate discussion about what information about CRC-screening would be needed to support their needs.

Session 3 in the female groups again focused on participants’ existing knowledge, this time about CRC-screening (first associations). In addition, based on how participants indicated they made decisions in session 2, we assessed what information about CRC-screening would be needed to support their needs. We asked them briefly about their decision-making process regarding CRC-screening. However, no extensive assignment was made on this. We evaluated reactions to various versions of the existing invitation letter/leaflet and to existing videos about the CRC-screening program. We also discussed information about performing the self-test, which is a fecal immunochemical test (FIT).

Session 3 in the male groups and session 4 in the female groups focused on concretizing novel information materials and strategies, based on insights derived from previous sessions. We provided participants with textual and visual information about the screening programs as prompts (Supplementary file 1, assignment 7 in female groups, assignment 5 in male groups) and asked them to select the most relevant information. Participants also completed a template in small groups about their ideas for group information sessions, for example time, location, who should provide the information (Supplementary file 1, assignment 6 in female groups, assignment 4 in male groups).

Background information was collected via two questionnaires. At the end of the first session, participants completed a questionnaire regarding their age, education level, place of birth of themselves and their parents, and self-perceived health literacy [41]. At the end of the third session, participants completed a questionnaire regarding their self-perceived numeracy [42] and self-reported previous participation in BC-screening (women) and CRC-screening (women and men). The questionnaires were developed for this study specifically, in Dutch. They were often filled in with support from researchers, intermediaries, or translators who could read the questions aloud and/or assist in filling in answers in case participants could not read or write. Supplementary file 4 provides English language versions of the questionnaires.

### Analyses

During the co-design sessions, one of the researchers took field notes and translated these into a summary afterwards. This was then checked and supplemented by the other researcher present, and when applicable, by the translator as well. Summaries of all sessions and assignments made were reviewed by three research team members (IvS, LS, AI). The researchers discussed their summaries together (16 meetings in total for the female sessions; 12 meetings in total for the male sessions) and presented their main findings to the project leader (OD). Summaries were also discussed with the citizen science board, one about the female sessions and one about the male sessions. Based on this, a thematic analysis was applied by three researchers each (IvS, LS, OD for female groups; IvS, AI, OD for male groups). In consensus meetings, all researchers presented their (initial) ideas about underlying themes, based on previous made summaries and discussions that had been held with the citizen science board. Together, key themes and issues related to the perspective and underlying needs of people from underserved groups regarding information provision were identified. This entire process was first conducted for the female groups and then for the male groups. Final overarching themes were then developed together.

## Results

### Participants

Table 2 displays the socio-demographic characteristics of the participants and their self-perceived health literacy and numeracy. As shown in the table, not all inclusion criteria were strictly followed and we allowed some flexibility. For example, we allowed one slightly younger woman to participate because she was part of a group that was already meeting in a community center. Likewise, in one group with women with a Moroccan background, two women without a migrant background participated.

**Table 2:** Participant characteristics

Group	No migrant background		Moroccan background		Turkish background		Surinamese background		Total
	Female groups	Male groups	Female groups	Male groups	Female groups	Male groups	Female groups	Male groups	
	*N* = 7	*N* = 7	*N* = 20 (13–19 per session)	*N* = 7	*N* = 9	*N* = 6	*N* = 8	*N* = 8	72
Age	53–78	61–78	44–71	54–71	53–72	54–74	40–70	56–78	Average age: (mn-max)
Low education ^1^ N (%)	6 (86%)	6 (86%)	12 (60%)	5 (71%)	9 (100%)	3 (50%)	2 (25%)	5 (63%)	
Low self-perceived health literacy N (%)	1 (14%)	1 (14%)	12 (60%)	4 (57%)	6 (67%)	4 (67%)	1 (13%)	1 (13%)	30 (42%)
Low self-perceived numeracy N (%)	4 (57%)	5 (71%)	13 (65%)	4 (57%)	5 (56%)	3 (50%)	5 (63%)	3 (38%)	42 (58%)
Participation in BC-screening N(%)	7 (100%)	n.a.	10 (3 missing data) (86%)	n.a.	7 (78%)	n.a.	6 (75%)	n.a.	30 (77%)
Participation in CRC-screening N (%)	6 (86%)	4 (57%)	6 (3 missing data) (35%)	4 (1 missing) (67%)	4 (44%)	4 (1 missing) (80%)	5 (1 missing) (71%)	3 (3 missing) (60%)	36 (57%)

^1^ Low education = Primary education, Pre-vocational secondary education, lower years of general secondary education, entry-level vocational training

### Perspective and underlying needs of people from underserved groups

A summary of objectives, methods, and results and key insights from all co-design sessions is provided in Supplementary file 2 (female groups) and Supplementary file 3 (male groups). The following three overarching themes were derived.

#### Theme 1: Limited knowledge about breast cancer, colorectal cancer and the idea of screening for these cancers, which made it difficult to relate cancer screening information in general to existing knowledge structures

In all groups, very limited knowledge existed about BC, CRC and cancer in general. Participants had many questions about the basics of cancer development and differences between cancer types, including their prevalence. For both cancer screening programs, but especially for CRC-screening, knowledge gaps and misconceptions were identified, with differences between gender and cultural groups. For example, among women with a Surinamese background, there were beliefs about the importance of taking care of one’s breasts, and that a mammogram would cause a ‘bad influence’ on breast tissue due to the compression of the breasts. These beliefs were not found in other female groups. The most prevailing misconception about CRC-screening was that a colonoscopy is the first test, which is not the case in the Netherlands. Another misconception was that screening would often be accompanied by a cancer diagnosis and that such diagnosis is almost invariably fatal. The role of polyps as a precursor to CRC and the idea of early detection was new to almost all participants. We also identified misconceptions about the FIT test, with people assuming that large amounts of stool would be needed and that other diseases could also be detected using the stool blood. Groups with a migration background indicated a need for information within the broader context of cancer prevention education, such as what people can do to reduce their risk of BC and CRC.

In general, people found the visual and numerical information that we provided (e.g., Supplementary file 1, assignment 4), interesting, but not key, except for explanations of the timeframe of invitations. Participants felt that this information could quickly be overwhelming and indicated that they needed explanation or ‘talk-throughs’, for example in a group information session.

#### Theme 2: People were in an early stage of the decision-making process, with positive but largely uninformed attitudes towards participation in BC- and CRC-screening

Most people were in an ‘early stage’ of a decision-making process, in which they were aware of BC-screening but not so much of CRC-screening, and were only vaguely aware of the rationale behind the screening programs. This was related to the lacking knowledge described in Theme 1. The groups were generally positive about both BC-and CRC-screening but had not actively thought about how participating or not would fit their own values. Their positive attitude appeared to be more based on general beliefs that cancer is a serious disease and cancer screening a good thing to do, although some people believed that attention to cancer could possibly bring cancer upon yourself.

When we did value clarification exercises with the groups, key values expressed mainly related to taking care of one’s health and body, also from a religious perspective, and the importance of family and being able to care for one’s family, the latter mainly in women. Men also emphasized the importance of being free from stress in daily life. Linking these values to whether or not to participate in cancer screening was new to all participants.

Participants showed limited engagement with information about potential benefits and harms of participation, which appeared to hinder their understanding. This was also related to the belief that screening was a diagnostic test performed when symptoms were present, and that screening would indicate the presence of cancer rather than precancerous stages (as with CRC-screening). Facts presented as potential benefits and harms in the Dutch leaflets such as ‘you can treat the cancer sooner’ (benefit), ‘you may get treatment when it is not needed’ (harm) and ‘cancer screening does not give you complete certainty’ (harm) were not actively reflected on in relation to their decisions. Participants especially stated that those harms were ‘disclaimer information only’ and they did not actively relate the harms (e.g., unnecessary tests/treatments) to previously expressed values, (e.g., the importance of being free of uncertainty or stress). Men without a migration background formed an exception to the other groups. They stressed that participation in CRC-screening might bring more harms than benefits to them. For example, they said that only limited extra survival would be gained, especially since they said to have other health problems and a relatively unhealthy lifestyle.

#### Theme 3: Participation in cancer screening was generally considered desirable, but at the same time not a priority in daily life

Participation in cancer screening was reported not to be a priority in daily life and invitations to be easily missed or forgotten, partly because participants had not actively considered participatation (related to Theme 2). Another aspect was that some participants did not fully understand how to make an appointment and that they did not consider it sufficiently important to ask others for help. Moreover, the belief that the screening test may be difficult to perform (CRC-screening) and the fact that they had to use digital methods (BC-screening) to make an appointment which they found complicated, were recurring themes in discussions. Participants stressed the need for creating awareness and emphasizing practical aspects, e.g., how to perform cancer screening tests, and the ease of performing the tests, especially the FIT test in CRC-screening, to increase the likelihood that decisions to participate will be implemented. In doing so, it was considered key that this information would be available in people’s native language and be culturally sensitive, and that reach out strategies would be employed, rather than just placing information on a website unknown or inaccessible. The groups with a migrant background proposed the idea of group information sessions in their local communities, to raise awareness, explain practical aspects and to provide opportunity to ask questions about cancer prevention and screening. This was believed to help them prioritize active decision-making.

### Implications for novel strategies to reach and inform underserved groups

**Fig. 2:**
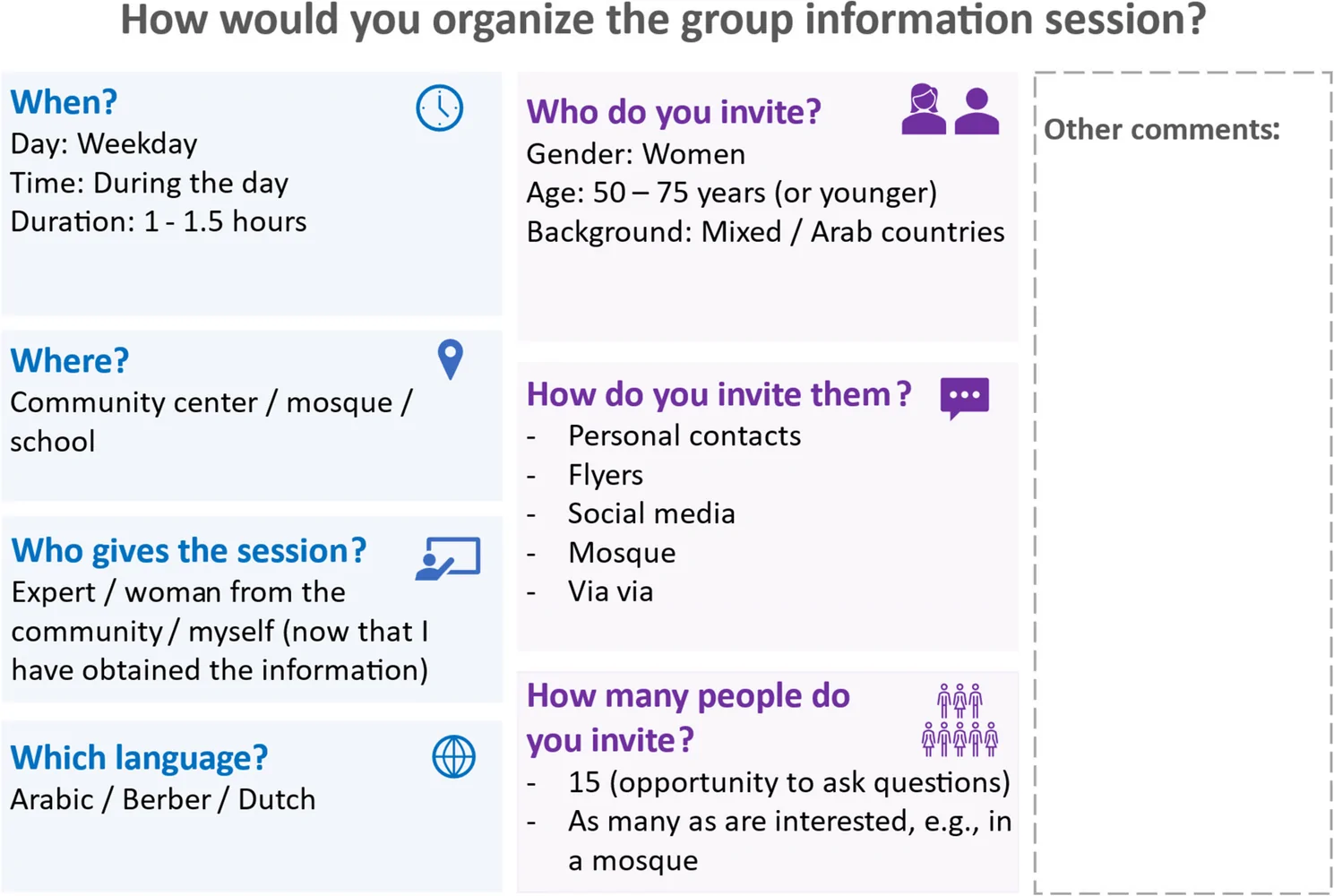
Example of template for form of group information sessions, composed based on the input derived from the women with a Moroccan background

**Fig. 3:**
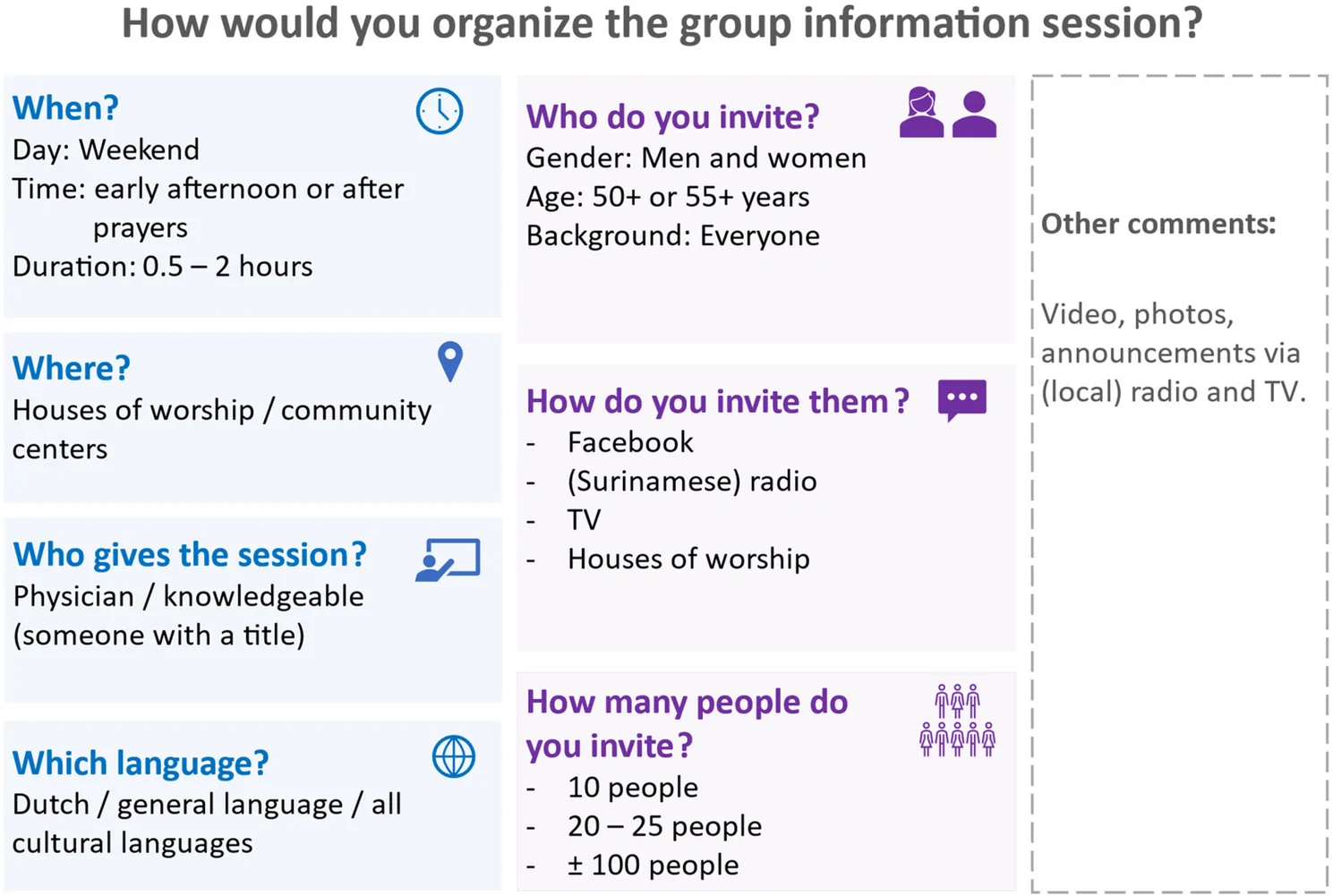
Example of template for form of group information sessions, composed based on the input derived from the men with a Surinam background

**Fig. 4:**
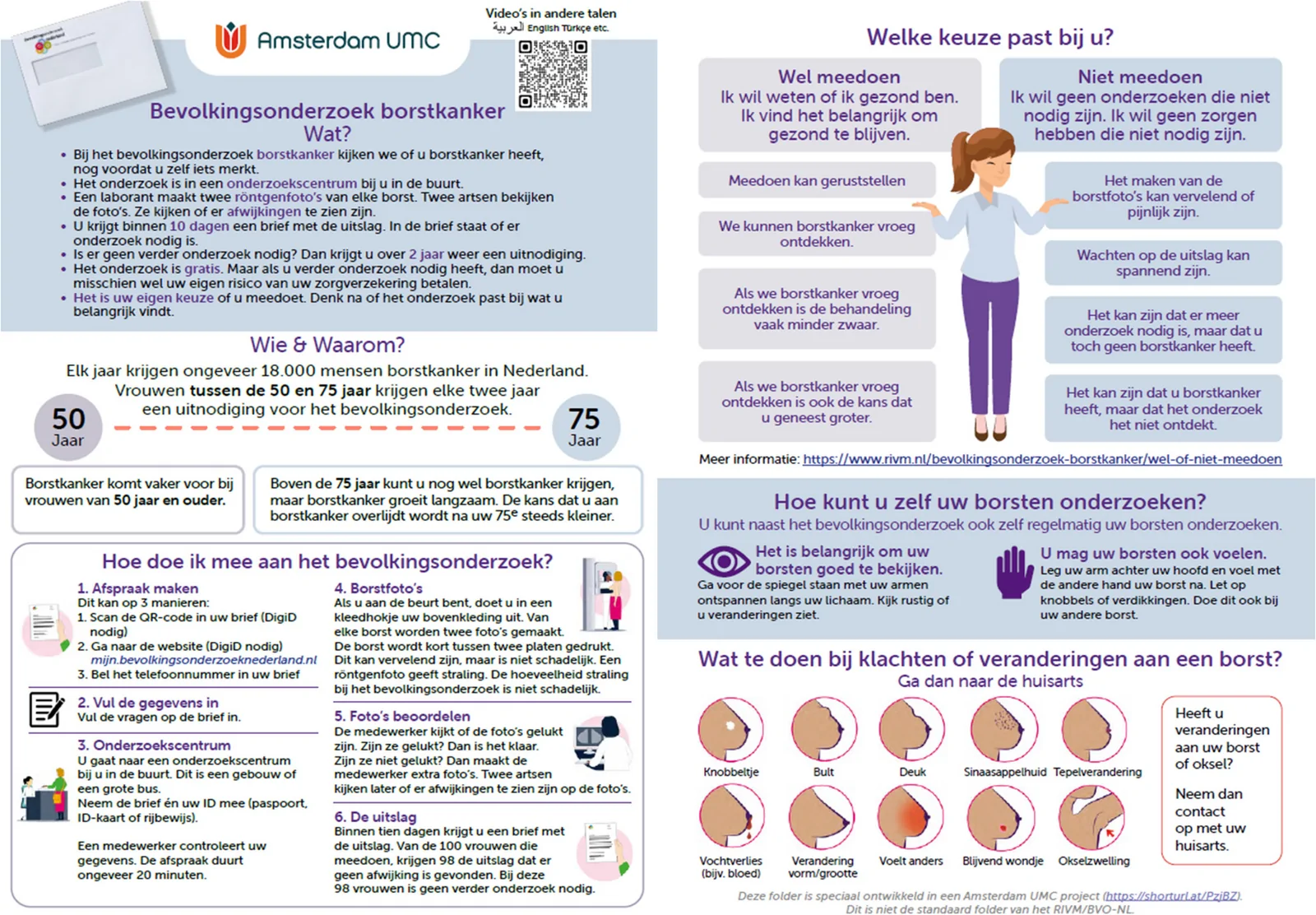
Co-created short awareness/information flyer about BC-screening (in Dutch)

**Fig. 5:**
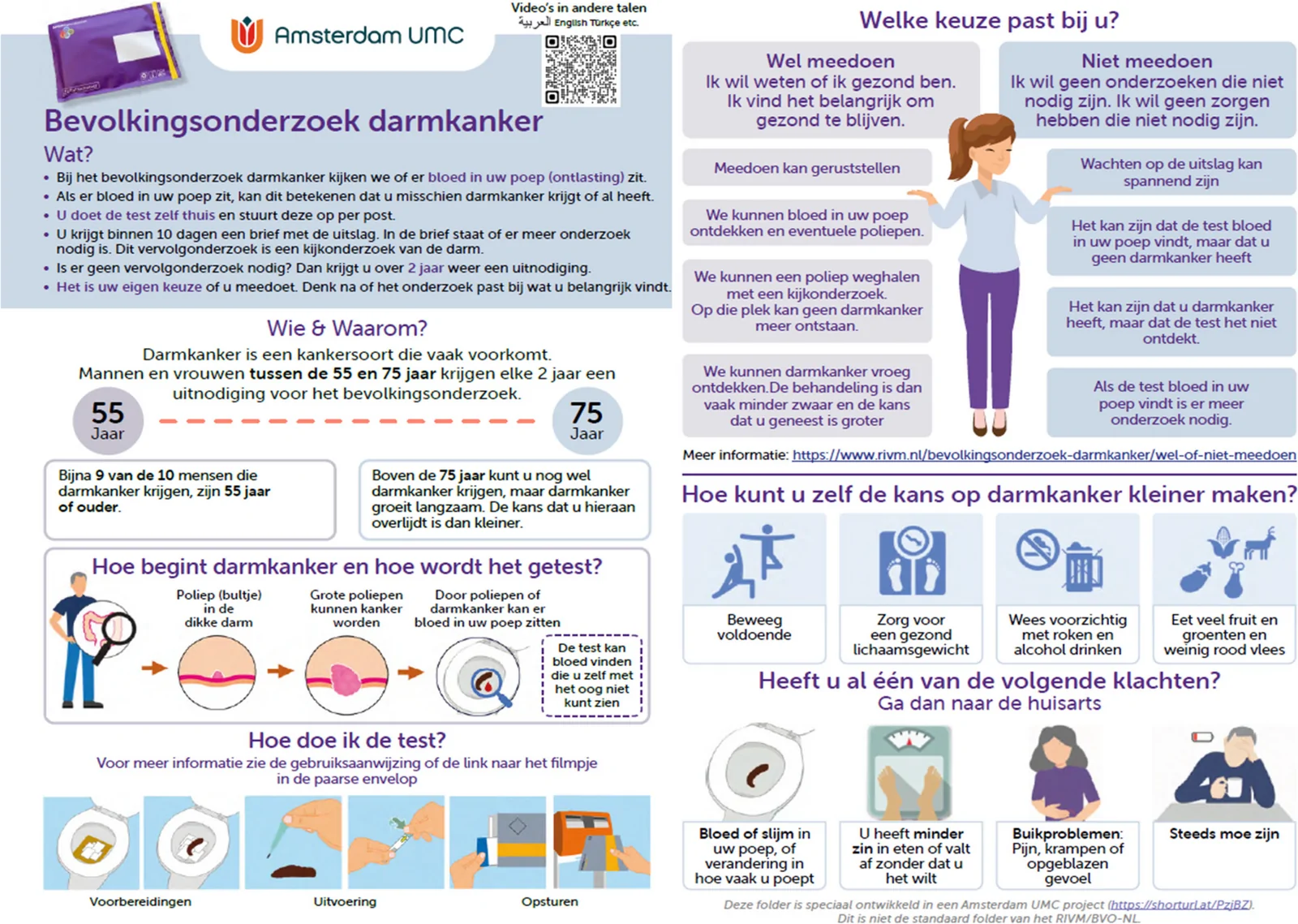
Co-created short awareness/information flyer about CRC- screening (in Dutch)

In the final sessions, participants worked on templates and content of potential novel information strategies. Three design directions were followed in all groups, based on results from earlier sessions: (1) group information sessions in local community settings; (2) short awareness/information flyers; (3) audiovisual information in native language. Together with participants, we designed templates for the form of group information sessions (Figs. 2 and 3 provide two examples), and short awareness/information flyers (Figs. 4 and 5). Audiovisual information in different languages was developed in another Dutch project parallel to our project. Based on our findings, we developed general guidelines regarding audiovisual information and we also provided specific recommendations for that project.

Based on the assignments, focal points for information content were formulated. First, information materials must take the very limited knowledge and the relatively early stage of the decision-making process into account. This implies that we should not start by explaining the test and listing potential harms and benefits, but ensuring that people first understand that different types of cancer exist and that screening programs use different types of screening tests. It must also be made crystal clear that the actual first tests in BC- and CRC-screening are meant for people without symptoms. Participants thought that this information could be best conveyed during group information sessions, where there is the opportunity to ask questions, or through short flyers. Information dissemination through social media was considered less suitable, because participants indicated difficulties to assess its reliability. However, certain groups appeared less interested in group information sessions. This was particularly true for men and women without a migrant background, and women with a Surinamese background- the latter specifically regarding BC- screening. Overall, these segments also demonstrated a stronger baseline knowledge, suggesting that alternative approaches might be more appropriate for them.

Second, a focus on the practical aspects of performing the tests is warranted. For BC-screening in the Netherlands, this would imply an explanation in lay language of how to make an appointment. For CRC-screening, the emphasis should be placed on performing the FIT test, immediately correcting the misconception that Dutch CRC-screening involves a colonoscopy as a primary test. Participants valued this information in audiovisual information in their native language, but emphasized that this could also be explained in short flyers and group information sessions. It was stressed that short flyers could be disseminated in local community centers and GP practices, reinforcing the idea that it is not complex or difficult to participate, while also serving as a reminder.

Third, although IDM might present complexities due to the limited knowledge among people from underserved populations and with attitudes merely guided by general beliefs (i.e. that cancer is serious and that cancer screening is a good thing to do), effort should still be made to foster more active decisions, rooted in people’s own values and priorities. While participants did not prioritize benefit/harm information themselves, they did report that they appreciated the value elicitation exercises, which were new to them. Participants said to welcome activities and discussions related to value clarification, especially during group information sessions. This suggests that there is room for more active, value-based decision-making regarding cancer screening participation.

## Discussion

This study aimed to investigate the perspective and underlying needs of people from underserved populations regarding information provision about BC- and CRC-screening in the Netherlands, and to design novel information strategies accordingly. The study was unique in that it was heavily grounded in co-design and guided by a citizen science board, and in involving a relatively large number of participants from underserved populations (*N* = 72), including those who were functional illiterate and/or illiterate. Moreover, we collaborated with them on improved products and strategies to support informed decision making (IDM), rather than only investigating the more well-known barriers in access to cancer screening. We found that people from underserved groups had very limited basic knowledge about the cancer types screened for, showed positive but largely uninformed attitudes towards participating in cancer screening, and struggled to prioritize cancer screening decisions in daily life, with invitations easily missed and forgotten.

The finding that people from underserved groups had limited basic knowledge, was also demonstrated in other studies [22, 29]. At the same time, both our study and other studies [22] found that people are eager to learn more about different types of cancer, cancer prevention, and the role of cancer screening. Especially our female participants indicated a preference for this information exchange through group information sessions in local community settings. On the one hand, such cancer education may offer opportunities to build cancer screening literacy in deprived urban neighborhoods. By addressing fundamental knowledge gaps and misconceptions, this approach can serve as a stepping stone for engaging individuals in screening decisions [35, 43]. On the other hand, it raises questions about how basic such information must be and whose responsibility it is to develop it. Cancer screening organizations may not necessarily consider it their responsibility to provide basic cancer education. In the sessions of the current study, a range of knowledge gaps and misconceptions were identified. To the best of our ability, we provided some basic education in the groups to resolve these misconceptions, and this was generally appreciated.

Questions remain about the content and feasibly of group information sessions providing, amongst others, such basic cancer prevention education. A first aspect is our finding that people without a migration background were not interested in group information sessions. Another concern is the potential for peer pressure during group sessions, which can hinder IDM. It is known that non-participation in cancer screening can be collectively constructed as bearing individual responsibility for the later development of cancer [44]. Other aspects are ethical and financial considerations, such as time and resources required to reach people from certain groups [37], which can compromise cost-effectiveness of screening programs.

Other focal points may be easier to address in information provision. For example, making crystal clear that BC- and CRC-screening are about early detection, that a bad result does not necessarily mean that you have cancer, especially in the case of CRC-cancer, and that cancer does not mean death. Although such issues may seem obvious for experts, they are not always emphasized explicitly in information materials. Misconceptions and fears, such as cancer screening being a diagnostic test for people with symptoms, that a bad result means cancer, and the belief that detection of cancer means death were also identified in studies in other countries [37, 45], suggesting widely held beliefs. Furthermore, translating key information materials into other languages, both text and audio, is a less intensive strategy with a potentially positive impact on cancer screening literacy. However, the question of how to disseminate such information is still open. Simply placing this information on a website will not suffice, as noted by our participants. Participants also indicated having difficulty assessing the reliability of information via social media, so distributing videos via social media does not seem to be an easy solution.

As in other studies [44, 46] participants were not that interested in information about potential harms of BC-and CRC-screening, and sometimes downplayed or rejected the information on harms. In most sessions, we offered some of this information in easy to understand formats, but participants understood it mainly as ‘disclaimer information’ that institutions are obliged to provide. These reactions were also demonstrated in a qualitative study among German and Turkish women in Germany [27]. This may suggest that population-based cancer screening and the rationale behind it, which are rather abstract epidemiological concepts, were not well-understood. However, reactions also seem to be related to a reliance on a dominant frame that cancer screening is a good thing to do. Scholars have previously argued that simply improving benefit/harm information may fail to engage people in cancer screening decisions, not only for people from underserved populations but more broadly [31, 44]. Performing value clarification exercises to reflect on aspects or goals people value may be a more productive strategy. Actively reflecting on aspects such as a need for reassurance or the wish to avoid unnecessary worry may provide more intuitive and affective meaning to abstract benefits and harms. Given that participants appreciated value clarification exercises, such assignments could help people from underserved groups make more active decisions about cancer screening.

### Study strengths and limitations

A strength of the study was that it was guided by a citizen science board, who helped us formulate focal points for the co-design sessions, recruitment and building trust among local communities. Their input was also used in the interpretation of findings. Another strength is that we reached people from the underserved groups and that we developed information prototypes together with them, instead of just formulating focal points for communication.

A limitation is that we only focused on one specific urban area, namely the Amsterdam region, and no other deprived areas of the Netherlands, and that the groups had relatively small sample sizes, which may limit generalizability. The Amsterdam region is characterized by a multicultural population, with many people with a non-Western migration background such as from Morocco, Turkey and Suriname. We are not sure if the results hold for other deprived urban areas or the more rural deprived areas of the Netherlands. In the more rural areas, -where fewer people with a migration background live- ‘being underserved’ in cancer screening may have a different meaning. We acknowledge that we may not have accounted for participation of nonbinary people. Although we did not explicitly require participants to join either the male or female groups, the distinction between male and female groups was made in practice and also advertised as such in recruitment materials. It might also be that people who participated in our sessions were relatively more interested in this kind of group activities. We may not have reached people who are more avoidant of group activities. A specific limitation related to the co-designed short awareness flyers may be that they contained explicit (visual) information about symptoms and that in case of symptoms, one should not wait for the cancer screening invitation but rather visit their GP directly. Although people found this important information, this could lead to people actually starting to connect cancer screening with symptoms in their minds.

## Conclusions

People from underserved populations in deprived urban areas of Amsterdam, the Netherlands, recognized the importance of BC- and CRC-screening in taking care of their health. However, some key information as currently provided by the Dutch screening organization was said to not come across. This was due to a mismatch between information content typically provided -focusing on cancer screening- and basic knowledge and information needs of the participants. There was also a mismatch with how the studied populations reported handling information in their daily lives. Participants in this study had not actively made a decision about cancer screening participation in the past. Co-designed strategies can be utilized by screening organizations to strengthen ‘cancer screening literacy’ and increase IDM.

## Supplementary Information

Supplementary Material 1.

Supplementary Material 2.

Supplementary Material 3.

Supplementary Material 4.

## Data Availability

Data and materials can be made available by the authors upon reasonable request (o.damman@amsterdamumc.nl).

## Abbreviations

BC-screening: Breast Cancer screening
CRC-screening: Colorectal Cancer screening
SES: Socio Economic Status
HL: Health Literacy
IDM: Informed Decision Making
