# Supplementary material for: Improving breast and colorectal cancer screening information through co-design with underserved groups in an urban area
Source: BMC Prim Care. 2026 Aug 19;27:374. doi: 10.1186/s12875-026-03522-z (PMC13681932; doi:10.1186/s12875-026-03522-z)
Supplement: Supplementary file 1 [file 12875_2026_3522_MOESM1_ESM.docx]

**Supplementary file 1: Key assignments and materials used in the different participatory design sessions (translated to English)**

Session 1 female groups

1. Assignment draft personas

2. Assignment values elicitation

Session 2 female groups

3. Assignment to think about the decision process and which thoughts, feelings, information, and opinions from others might influence the decision about BC-screening

1.

4. Various versions of existing information shown to participants to stimulate discussion about what information about CRC-screening would be needed to support needs

Session 3 female groups

5. Assignment to think about the decision process and which thoughts, feelings, information, and opinions from others might influence the decision about CRC-screening

Session 4 female groups

6. Assignment development group information sessions

7. Prompts with different types of information about CRC-screening to prioritize information for a short leaflet

Session 1 male groups

1. Assignment draft personas

2. Assignment value elicitation

Session 2 male groups

3. Assignment to think about the decision process and which thoughts, feelings, information, and opinions from others might influence the decision about CRC-screening

Session 3 male groups

4. Assignment development group information sessions

5. Prompts with different types of information about CRC-screening to prioritize information for a short leaflet
