# Supplementary material for: Improving breast and colorectal cancer screening information through co-design with underserved groups in an urban area
Source: BMC Prim Care. 2026 Aug 19;27:374. doi: 10.1186/s12875-026-03522-z (PMC13681932; doi:10.1186/s12875-026-03522-z)
Supplement: Supplementary file 2 [file 12875_2026_3522_MOESM2_ESM.docx]

Supplementary file 2: Summary of the objectives, methods, results, and key insights from all participatory sessions with female participants

| **Session and objective** | **Methods and assignments uses** | **Results** | **Notable differences between groups** | **Key insights for information provision** |
| --- | --- | --- | --- | --- |
| Session 1 (BC-screening): Getting to know the target group, identifying key values in daily life and with regard to health, and gaining insight into the information channels used. | A template with a persona and the assignment to further develop this persona with us. | Participants all expressed the importance of health and spending time with others such as family & friends. All groups emphasized getting up early to get all things done. Frustrations in daily life included disrespect or negative contact with others, not knowing how to arrange things and being tired after all daily duties.  Participants said to obtain information from a variety of sources such as local community centers, GPs, family & friends, TV/radio and social media. | Women with a Moroccan background emphasized their life goal of praising Allah and visiting Mekkah. | Information has to fit within a full schedule of daily tasks.  Information should emphasize how to perform and arrange things easily and not be too overwhelming in the first place. |
|  | Value elicitation methods | Participants enjoyed doing these exercises, often emphasizing that this was the first time they actively considered their values in such a way. Participants indicated that they had not previously considered how participation or non-participation in BC-screening fits with these values or priorities.  Participation in BC-screening seemed to fit overall with expressed values/priorities. | Women with Moroccan/Turkish background emphasized health, taking care of oneself and family, and faith  Women with a Surinamese/no migration background emphasized health, independence, my body/my decision | Information should encourage women to think more actively about the fact that there is a choice to be made based on their values, instead of overwhelming them with an abstract list of potential benefits and harms. |
|  | Group discussion on how participants deal with information from various sources in daily life, based on a post-it exercise, and discussions after a prompt with an invitation envelope for BC-screening. | Participants emphasized the role of trust in the provider of information. TV/radio (from the Netherlands or their home country) are also perceived as trusted sources for medical information.  Participants stressed the ease of information exchange through social media, but also the difficulty of assessing reliability and find back information.  The invitation envelopes were received with mixed reactions. Some emphasized the importance of a trusted ‘logo’ and others the term ’health’ on the envelope. | Women without a migration background said to not visit community centers.  Women with a Turkish background said to not obtain information form their GP. | It is important that the same information comes repeatedly through different sources, so that the invitation for BC-screening is recognized. |
|  | Group discussion about first associative knowledge about BC-screening | Participants were generally aware of the BC-screening program and had some basic knowledge, mostly from their mothers or other women. Most were positive about participating but pain during the mammography was a recurrent theme of discussion.  Participants expressed some confusion about changes in the program that were made since its start in the 1970’s, especially regarding the invitation procedure, making an appointment and the screening locations. | Some women with a Moroccan background and some with a Surinamese background believed that paying attention to cancer could potentially bring cancer upon themselves  Women with a Surinamese background emphasized the importance of taking good care of one’s breasts, e.g. teaching daughters not to play too rough. | Rather than stating that a mammography can be ‘uncomfortable’, information could state more clearly that it can be painful.  It could be useful to explicitly state in information whether there are changes to the BC-screening program compared to the previous time women participated. |
| Session 2 (BC-screening): Exploring/defining how decisions are made, what knowledge already exists about BC-screening, and what information is needed to support needs in BC-screening | Templates completed in pairs about the decision process. | Participants expressed basic knowledge about BC-screening, but only a vague understanding of the rationale behind the screening program. It was often assumed that mammography was intended as a diagnostic test for symptoms.  Participants predominantly expressed positive thoughts and feelings about participation in BC-screening. Non-participation was linked to being scared or worried.  Participation was not seen as a priority in daily life. The fact that they had to make their own appointments online was a recurring issue of discussion. | Women without a migration background emphasized that the information leaflet would not play a serious role in their decision-making process; they said to follow their feelings and beliefs. | Importance of improving understanding the essence of BC- screening, such as the 'what,' 'for whom,' and 'why'. Practical aspects, such as how to make an appointment, should be clearly explained. |
|  | Group discussion about potential benefits and harms of participating in BC-screening. | Participants mostly saw benefits, only a few participants expressed to perceive potential harm such as (unnecessary) worry. Information about potential benefits and harms was often only experienced as 'disclaimer information'. | Migrant groups expressed gratefulness that BC-screening exists, and emphasized the benefits  Women with a Surinamese background expressed beliefs about the harmfulness of a mammography (bad for your breasts) | Importance of encouraging women to think more actively about the fact that there is a choice to be made, instead of overwhelming them with an abstract list of potential benefits and harms.  Relating potential benefits and harms to important personal values in decision-making. |
|  | Discussing different existing visuals and audiovisual information used for the BC-screening program. | Participants were positive about the visual examples shown and generally said to prefer them over textual information.  Audiovisual information was said to be easily listened to and disseminated through personal contact (e.g., in texting each other), once received. | No notable differences | Importance of getting audiovisual information in one’s mother language at the target groups in the first place.  Importance of visual information in the invitation leaflets |
| Session 3 (CRC-screening): Assessing existing knowledge about CRC-screening and what information is needed to support needs in CRC-screening | Prompts with versions of existing information shown to participants to stimulate discussion. | Participants had less knowledge or awareness about CRC-screening and were at a relatively early stage of the decision-making process. Generally, participants were positive about the program, but less pronounced compared to BC-screening.  Participants showed misconceptions about what the self-test (FIT) entailed, such as the idea that it involves a large amount of stool | Women with a migrant background expressed fear and worry about performing the self-test (FIT) in the CRC-screening program. | Importance of improving understanding the essence of CRC- screening, such as the 'what,' 'for whom,' and 'why'.  Improve awareness /understanding that the primary test in CRC-screening is a self-test of a small stool sample and not a colonoscopy.  Importance of explaining the (ease) of performing the FIT test in the CRC-screening program. |
| Session 4 (CRC-screening): Concretizing novel information materials and strategies, based on the insights derived from the previous sessions | Template for development group information sessions | Participants were overall positive about group information sessions, which arose spontaneously, similar to the idea of meeting each other in the participatory design sessions. | Women without a migration background and women with a Surinamese background did not prefer group information sessions in local community centers for BC-screening, but they were more open to it for CRC-screening. | Importance of a combined strategy. |
|  | Prompts with different types of information about CRC-screening to prioritize information for a short leaflet | Participants expressed the importance of both ‘leaflet information’ and audiovisual information in one’s mother language. They created draft leaflets with the key information for themselves, emphasizing that CRC-screening exists and what it entails. | No notable differences | Importance of being an ‘awareness flyer’ in the first place, with more layers of information for those who want to read more.  Importance of audiovisual information on one’s mother language.  Emphasis on the ease of the FIT test and that it is not dirty to perform the FIT test. |
