# Supplementary material for: Improving breast and colorectal cancer screening information through co-design with underserved groups in an urban area
Source: BMC Prim Care. 2026 Aug 19;27:374. doi: 10.1186/s12875-026-03522-z (PMC13681932; doi:10.1186/s12875-026-03522-z)
Supplement: Supplementary file 3 [file 12875_2026_3522_MOESM3_ESM.docx]

Supplementary file 3: Summary of the objectives, methods, results, and key insights from all participatory sessions with male participants

| **Session and objective** | **Methods and assignments uses** | **Results** | **Notable differences between groups** | **Key insights for information provision** |
| --- | --- | --- | --- | --- |
| Session 1 (CRC-screening): Getting to know the target group and elicitation key values in daily life as well as related to health, and gaining insight into the information channels used. | A template with a persona and the assignment to further develop this persona with us. | Participants generally expressed the importance of health, being active and free of stress in life, and wanting a good life for their children.  Most men said to enjoy being active during the day, doing chores, and watching sports on TV. Some men said to have relatively unhealthy lifestyle  Frustrations in daily life mentioned were ‘negativity’ in society, and, for some men, difficulties with reading, writing and/or speaking Dutch, and with digital activities.  Participants said to obtain information from internet, social media, TV and from family & friends. | Men with a Moroccan background emphasized religious goals and the importance of asking help if needed.  Men with a Surinamese background emphasized societal participation and contribution.  Men without a migration background said to not meet regularly with other men.  Men with a Turkish background and men without a migration background expressed some distrust against institutions and experienced that institutions don’t care for them. | Information has to fit different styles of information access, from active men in community centers to more passive men not going out that much.  Information has to take into account existing distrust in institutions among some men.  Information must be at a basic reading level in Dutch. (In Dutch this is called ‘B1 level’ which is equivalent to Low-Intermediate / Intermediate level for American English or equivalent to Cambridge Preliminary / PET level for British English). |
|  | Value elicitation methods | Participants found it difficult to do these exercises. Participants indicated that they had not previously considered how participation or non-participation in CRC-screening fits with these values or priorities.  Participation in CRC-screening seemed to fit overall with expressed values/priorities such as health and spending time with family, although it was at odds with the importance attached to being free of stress during daily life. | Men with Moroccan, Turkish and Surinamese background emphasized health, family, enjoying life, being free of stress.  Faith and religion were important to men with a Moroccan and Turkish background. | Information should encourage men to think about CRC-screening in relation to their values and priorities. |
|  | Group discussion on how participants dealt with information from various sources in daily life based on a post-it exercise, and discussions after a prompt with an invitation envelope for BC-screening. | Participants emphasized the role of good command of the Dutch language, which was lacking in some groups, also in men without a migration background.  Some men actively gained health related information from media in their country of origin.  The invitation envelopes were received with mixed reactions. Some emphasized the importance of a trusted ‘logo’ and others the term ’health’ on the envelope. | Men with a Surinamese background emphasized being active in information search from different sources, from a societal engagement.  Men with no migration background expressed difficulties with reading and writing and showed some distrust against institutions and authorities. | It is important that the same information comes repeatedly through different sources, so that the invitation for CRC-screening is recognized and trusted. |
|  | Group discussion about first associative knowledge about CRC-screening | Participants were generally unaware of the CRC-screening program and said to be scared/worried of a potential bad result, which would indicate cancer.  Overall, men were positive about the existence of the CRC-screening program from a societal perspective.  Participants expressed some confusion about colorectal cancer in relation to other types of cancer. | Men without a migration background emphasized their relatively unhealthy lifestyle as a potential reason not to participate in CRC-screening  Men with a Moroccan and a Turkish background emphasized a taboo on cancer and a worry about a potential bad result. | It is important to emphasize the purpose of CRC screening, which is not only to detect cancer but also to prevent cancer from developing. This means that if an abnormality is detected, it may indicate the presence of a polyp rather than cancer itself. |
| Session 2 (CRC-screening): Exploring/defining how decisions are made, what knowledge already exists about CRC-screening, and what information is needed to support needs in CRC-screening | Templates completed in pairs about the decision process. | Participants expressed limited knowledge about CRC-screening, and compared it to prostate cancer screening. It was often assumed that a colonoscopy was the first test, that this is a diagnostic test for people with symptoms, and that a bad result means that you will die.  Participants predominantly expressed positive thoughts and feelings about participation in CRC-screening, especially after explaining the FIT test.  There were questions about the cessation age of 75 years and why CRC-screening is not continued.  Participation was not seen as a priority in daily life. The fact that the current information is too lengthy to read and that the FIT test may be difficult to perform were recurrent themes. | Men with a Turkish and background and those with a Moroccan background asked many questions about cancer and CRC-cancer, e.g., what is cancer, what are complaints, what can I do to prevent cancer?  There was more basic knowledge in those with a Surinamese background and those without a migration background. | Importance of improving understanding the essence of CRC- screening, such as the 'what,' 'for whom,' and 'why’.  Importance of explaining the idea of early detection, the role of polyps, that a bad result does not mean that you’ll die, and that blood in the stool does not automatically mean cancer.  Importance of clearly explaining how to do the FIT test, and emphasizing that it is not difficult.  Information should be as concise as possible, and not a ‘complete package’. |
|  | Group discussion about potential benefits and harms of participating in CRC-screening. | Participants mostly saw benefits, men also talked about the wish to avoid (unnecessary) worry.  However, men did not seem to actively consider benefits and harms in decision-making.  Information about potential benefits and harms was often only experienced as 'disclaimer information'. | In men with a Moroccan and a Turkish background, there was a discussion about the potential contagiousness of cancer and that others might avoid you when you receive a bad result from CRC-screening.  Men without a migration background emphasized the importance of communicating that a potential follow-up test does involve costs (‘eigen Risico’ in Dutch) | Importance of encouraging men to think more actively about the fact that there is a choice to be made, instead of overwhelming them with an abstract list of potential benefits and harms.  One key ‘benefit’ to emphasize seems to be the idea of early detection/screening and that this is different from a diagnostic test of cancer.  Relating potential benefits and harms to important personal values in decision-making. |
|  | Discussing different existing visuals and audio visual information used for the BC-screening program. | Participants were positive about shown examples, especially those explaining the process of CRC-screening.  Audiovisual information was said to be easily disseminated through personal contact (e.g., in texting each other), once received.  The audiovisual information on how to perform the stool test caused positive reactions/relief. | Men with a Turkish background emphasized the importance of information being available in Turkish. | Importance of getting audiovisual information in one’s mother language at the target groups in the first place. |
| Session 3 (CRC-screening): Concretizing novel information materials and strategies, based on the insights derived from the previous sessions | Template for development group information sessions | Participants were overall positive about group information sessions, that spontaneously came up, similar to the idea of meeting each other in the participatory design sessions. | Men without a migration background did not prefer group information sessions in local community centers. They said it might work for others, but not for them. | Importance of a combined strategy. |
|  | Prompts with different types of information about CRC-screening to prioritize information for a short leaflet | Participants expressed the importance of both ‘leaflet information’ and audiovisual information in one’s mother language. They created draft leaflets with the key information for themselves, emphasizing that CRC-screening exists and what it entails, especially an explanation of the procedure of the stool test. | No notable differences | Importance of being an ‘awareness flyer’ in the first place, with more layers of information for those who want to read more. Emphasis on that screening is for those without complaints. |
