# Supplementary material for: Improving breast and colorectal cancer screening information through co-design with underserved groups in an urban area
Source: BMC Prim Care. 2026 Aug 19;27:374. doi: 10.1186/s12875-026-03522-z (PMC13681932; doi:10.1186/s12875-026-03522-z)
Supplement: Supplementary file 4 [file 12875_2026_3522_MOESM4_ESM.docx]

Supplementary file 4: Questionnaires translated to English

Questionnaire 1 (filled in after session 1)

**Amsterdam UMC Study:
Improving Information About Breast Cancer and Colorectal Cancer Screening**

1. How old are you? …………… years old
2. Are you male or female?
   ⃝ Male
   ⃝ Female
3. What is your highest level of education completed?
   ⃝ No formal education
   ⃝ Primary school / Elementary education
   ⃝ Lower secondary education (VMBO)
   ⃝ Upper secondary education, first 3 years (HAVO / VWO lower cycle)
   ⃝ Upper secondary education, completed (HAVO / VWO)
   ⃝ Vocational education, level 1 (MBO 1)
   ⃝ Vocational education, levels 2 to 4 (MBO 2-4)
   ⃝ Associate degree (2-3 year higher professional education)
   ⃝ Bachelor's degree (HBO or University)
   ⃝ Master's degree / Doctorate (HBO or University)
4. In which country were you born?
   ⃝ The Netherlands
   ⃝ Turkey
   ⃝ Morocco
   ⃝ Suriname
   ⃝ Other, please specify: ……………………………………..
5. In which country was your mother born?
   ⃝ The Netherlands
   ⃝ Turkey
   ⃝ Morocco
   ⃝ Suriname
   ⃝ Other, please specify: ……………………………………..
6. In which country was your father born?
   ⃝ The Netherlands
   ⃝ Turkey
   ⃝ Morocco
   ⃝ Suriname
   ⃝ Other, please specify: ……………………………………..
7. Which language do you speak most frequently at home?
   ⃝ Dutch
   ⃝ Other language

**The following questions focus on how you manage health-related information.**

1. How often do you have someone help you read hospital materials?

⃝ Never
⃝ Occasionally
⃝ Sometimes
⃝ Often
⃝ Always

2. How confident are you filling out medical forms by yourself?
   ⃝ Not at all

⃝ A little bit

- Somewhat
  ⃝ Quite a bit
  ⃝ Extremely

3. How often do you have problems learning about your medical condition because of difficulty understanding written information?
   ⃝ Never
   ⃝ Occasionally
   ⃝ Sometimes
   ⃝ Often
   ⃝ Always

Questionnaire 2 (filled in after session 3)

**Amsterdam UMC Study:
Improving Information About Breast Cancer and Colorectal Cancer Screening**

1. **How good are you at doing the following things?**

| a. | | How good are you at working with fractions? | | | | | | | | | | | |
| --- | --- | --- | --- | --- | --- | --- | --- | --- | --- | --- | --- | --- | --- |
|  |  | | |  | | |  | |  | |  | |  |
|  | | 1 | 2 | | 3 | 4 | | 5 | | 6 | |  | |
|  |  | 🞏 | 🞏 | | 🞏 | 🞏 | | 🞏 | | 🞏 | |  | |
|  |  | **Not at all good** |  | |  |  | |  | | **Extremely good** | |  | |
|  | |  | | | | | | | | | | | |
| b. | | How good are you at working with percentages? | | | | | | | | | | | |
|  |  | | |  | | |  | |  | |  | |  |
|  | | 1 | 2 | | 3 | 4 | | 5 | | 6 | |  | |
|  |  | 🞏 | 🞏 | | 🞏 | 🞏 | | 🞏 | | 🞏 | |  | |
|  |  | **Not at all good** |  | |  |  | |  | | **Extremely good** | |  | |
|  | |  | | | | | | | | | | | |
| c. | | How good are you in calculating a 5% tip? | | | | | | | | | | | |
|  |  | | |  | | |  | |  | |  | |  |
|  | | 1 | 2 | | 3 | 4 | | 5 | | 6 | |  | |
|  |  | 🞏 | 🞏 | | 🞏 | 🞏 | | 🞏 | | 🞏 | |  | |
|  |  | **Not at all good** |  | |  |  | |  | | **Extremely good** | |  | |
|  | |  | | | | | | | | | | | |
| d. | | How good are you at figuring out how much a shirt will cost if it is 25% off? | | | | | | | | | | | |
|  |  | | |  | | |  | |  | |  | |  |
|  | | 1 | 2 | | 3 | 4 | | 5 | | 6 | |  | |
|  |  | 🞏 | 🞏 | | 🞏 | 🞏 | | 🞏 | | 🞏 | |  | |
|  |  | **Not at all good** |  | |  |  | |  | | **Extremely good** | |  | |

2. **Please check the box that best reflects your answer**

| e. | | When reading the newspaper, how helpful do you find tables and graphs that are parts of a story? | | | | | | | | | | | |
| --- | --- | --- | --- | --- | --- | --- | --- | --- | --- | --- | --- | --- | --- |
|  |  | | |  | | |  | |  | |  | |  |
|  | | 1 | 2 | | 3 | 4 | | 5 | | 6 | |  | |
|  |  | 🞏 | 🞏 | | 🞏 | 🞏 | | 🞏 | | 🞏 | |  | |
|  |  | **Not at all helpful** |  | |  |  | |  | | **Extremely helpful** | |  | |
|  | |  | | | | | | | | | | | |
| f. | | When people tell you the chance of something happening, do you prefer that they use **words** (“it rarely happens”) or **numbers** (“there is a 1% chance”)? | | | | | | | | | | | |
|  |  | | |  | | |  | |  | |  | |  |
|  | | 1 | 2 | | 3 | 4 | | 5 | | 6 | |  | |
|  |  | 🞏 | 🞏 | | 🞏 | 🞏 | | 🞏 | | 🞏 | |  | |
|  |  | **Always prefer words** |  | |  |  | |  | | **Always prefer numbers** | |  | |
|  | |  | | | | | | | | | | | |
| g. | | When you hear a weather forecast. Do you prefer predictions using **percentages** (e.g., “there will be a 20% chance of rain today”) or predictions using only **words** (e.g., “there is a small chance of rain today”)? | | | | | | | | | | | |
|  |  | | |  | | |  | |  | |  | |  |
|  | | 1 | 2 | | 3 | 4 | | 5 | | 6 | |  | |
|  |  | 🞏 | 🞏 | | 🞏 | 🞏 | | 🞏 | | 🞏 | |  | |
|  |  | **Always prefer percentages** |  | |  |  | |  | | **Always prefer words** | |  | |
|  | |  | | | | | | | | | | | |
| h. | | How **often** do you find numerical information to be useful? | | | | | | | | | | | |
|  |  | | |  | | |  | |  | |  | |  |
|  | | 1 | 2 | | 3 | 4 | | 5 | | 6 | |  | |
|  |  | 🞏 | 🞏 | | 🞏 | 🞏 | | 🞏 | | 🞏 | |  | |
|  |  | **Never** |  | |  |  | |  | | **Very often** | |  | |

3. **Have you received an invitation for the breast cancer screening program?**

⃝ Yes
⃝ No -> you can skip question 4

4. **Have you participated in the breast cancer screening program?**

⃝ Yes, every time
⃝ Yes, but not every time
⃝ No

5. **Have you received an invitation for the colorectal cancer screening program?**

⃝ Yes
⃝ No -> you can skip question 6

6. **Have you participated in the colorectal cancer screening program?**

⃝ Yes, every time
⃝ Yes, but not every time
⃝ No
